# Impact factors 2015; NHJ on the rise!

**DOI:** 10.1007/s12471-016-0870-2

**Published:** 2016-08-02

**Authors:** E. E. van der Wall

**Affiliations:** Netherlands Society of Cardiology/Holland Heart House, Utrecht, The Netherlands

In June 2016, the impact factors for the year 2015 were released (Thomson Reuters Journal Citation Reports® JCR 2015, ISI Web of Knowledge). The 2015 impact factor of the Netherlands Heart Journal (NHJ) amounted to 2.062, showing a clear rise compared with the 2014 impact factor (1.837).

The impact factor is defined as the average number of citations received per paper divided by the published articles in a specific journal during the preceding two years [1, 2, 3]. The 2015 NHJ impact factor was calculated as follows: in 2015 the total number of *citations* to articles published together in the years 2013 and 2014 amounted 301. The total number of *articles* published together in 2013 and 2014 was 132. As a result, the 2015 impact factor was 301 citations divided by 132 articles: 301/132 = 2.062. NHJ received its first impact factor in 2009, which was 1.392 at that time; it increased to 2.263 in 2013, decreased in 2014 but now appears to be back on track again with 2.062 [4, 5].

How does NHJ stand internationally? Out of a total number of 124 JCR-listed cardiovascular journals (*subject category Cardiac & Cardiovascular Systems*) NHJ takes place 68 on the list of impact factors for 2015, being therefore in the midrange. This can be considered to be rather good for a national journal from a relatively small country. As is well known, the top-3 cardiovascular journals – already for quite some years now – are the Journal of the American College of Cardiology (JACC), Circulation, and the European Heart Journal (EHJ). The 2015 impact factor for JACC was 17.759, for Circulation 17.047, and for EHJ 15.064. However, the ranking order of the top-3 journals may vary each year.

At the political level, NHJ participates in the HEART Group (Heart Editors Action Round Table), consisting of a group of Editors-in-Chief of about 40 leading international cardiovascular journals worldwide in order to discuss areas of growing common interest, such as ethical issues including scientific fraud, plagiarism, and double publication. [6]. The group also addresses statements on matching languages to ‘match’ the type of study conducted [7]. The Heart Group convenes annually at the three major Cardiology Congresses, namely the ACC, ESC and AHA meetings.

How does NHJ stand at the European level? Several years ago, the European Society of Cardiology (ESC) launched the Editors’ Network with the purpose to promote the dissemination and implementation of high-quality editorial standards among the ESC National Societies Cardiovascular Journals (NSCJ) [8]. The Editors’ Network consists of the Editors-in-Chief of all ESC-connected NSJC. The main aims of the Editors‘ Network are to foster collaboration between the national societies and the ESC by diffusing scientific content, promoting editorial excellence, streamlining review processes, and addressing ethical issues and conflicts of interests [9, 10, 11]. Currently there are 56 ESC-related countries that together have 60 national society journals (some countries have more than one national society journal). Of those national society journals, only 11 (18 %) have an impact factor; of those 11 journals NHJ currently takes the sixth place, something to be truly proud of. The Editors’ Network gathers twice a year, once at the Spring meeting in the European Heart House and once at the annual ESC Congress.

Over time, impact factors have become the holy grail in the scientific journal domain [12]. Many authors wish to publish in journals with the highest impact factors, because it will increase their scientific image, their professional profile, and their academic career perspectives. As a result, every journal editor works hard to improve the journal‘s impact factor because it is viewed by publishers as an index of journal quality and success, determining the extent to which the journal is resourced by its sponsoring organisation or publisher. Despite proposals for other parameters of journal quality, such as *article-level metrics*, the impact factor is still considered to be the *nec plus ultra* for authors, editors, publishers, and academic institutions [13, 14]. Therefore, one still remains dependent on the impact factor for a journal quality index.

Lastly, we wholeheartedly thank the authors of the papers for sending their fine work to NHJ and we are grateful to the Editorial Board members for carefully reviewing the manuscripts. We would also like to acknowledge the support of our national society, the Netherlands Society of Cardiology. Of course, will continue to strive to increase our impact factor over the forthcoming years! [15].
